# A Short Report of Mental Health Issues among the Young people: Insight from the Fasa Cohort Study: Mental Health Issues Among the Young people based on Fasa Cohort Study

**DOI:** 10.31661/gmj.v14i.3797

**Published:** 2025-07-01

**Authors:** Mohammad Saeed Soleimani Meigoli, Bahareh Fakhraei, Nematollah Jaafari, Azizallah Dehghan, Mojtaba Farjam

**Affiliations:** ^1^ Student Research Committee, Fasa University of Medical Sciences, Fasa, Iran; ^2^ Department of Psychiatry, School of Medicine, Fasa University of Medical Sciences, Fasa, Iran; ^3^ Université de Poitiers, Centre de Recherche sur la Cognition et les Apprentissages CNRS 7295, Unité de Recherche Clinique en Psychiatrie du Centre Hospitalier Henri Laborit 86000, Poitiers, France; ^4^ Noncommunicable Diseases Research Center, Fasa University of Medical Sciences, Fasa, Iran; ^5^ Clinical Research Development Unit, Valiasr Hospital, Fasa University of Medical Sciences, Fasa, Iran

**Keywords:** Youth Mental Health, Depression and Anxiety, Risk Behaviors, Cohort Study

## Dear Editor,

**Table:** Table1. Logistic Regression Results for Predictors of Depression and Anxiety

**Variable**		**Depression**			**Anxiety**	
	**OR(Exp(B))**	**[95% CI]**	**Sig.**	**OR(Exp(B))**	**[95% CI]**	**Sig.**
Gender	1.563	[1.268-1.927]	<0.001	1.452	[1.156-1.825]	0.001
Marital Status	0.679	[0.511-0.901]	0.007	0.84	[0.615-1.147]	0.27
Employment Status	0.598	[0.474-0.754]	<0.001	0.53	[0.413-0.681]	<0.001
Body Mass Index	1.223	[0.918-1.628]	0.16	1.392	[1.041-1.860]	0.02
Age	1.186	[1.069-1.316]	0.001	1.483	[1.320-1.665]	<0.001
Opium Use	3.593	[2.868-4.501]	<0.001	2.556	[2.005-3.259]	<0.001
Alcohol Use	1.454	[0.983-2.150]	0.06	1.969	[1.311-2.957]	0.001
Smoking	1.504	[1.184-1.906]	0.001	1.434	[1.109-1.854]	0.006
Constant	0.025		<0.001	0.021		<0.001

**Exp(B):** Exponential of the Beta Coefficient (Odds Ratio); **OR:** Odds Ratio; **CI:** Confidence Interval; **Sig.:** Significance Level (P-value)

Particularly for teenagers and young adults, anxiety, depression, and other mental health conditions continue to be major public health concerns [1]. These transitory phases bring about significant changes in a person’s social, emotional, and physical makeup, which may make them more susceptible to mental health problems. The serious long-term consequences of ignoring these issues highlight how important it is to act quickly [2].

The Fasa Youth Cohort project was a comprehensive epidemiological study designed to ascertain the prevalence of mental health disorders and their relationship to some of risky behaviors in young Iranians. A study with 3,013 participants aged 15-35 years used validated methods and standardized questionnaires (CIDI 2.1) to identify those who experienced mental health problems in the past year and obtain reliable data [3][4].

Results related to mental health were linked using regression analysis as presented in Table-1. The study found that 16.9% of participants had anxiety and 20.7% had depression. Females had higher rates of anxiety (19.9%) and depression (24.1%) than males (12.7% and 16.1%); widowed people had the highest rates of anxiety (50%) and depression (58.3%). Women had a significantly greater risk of depression (OR = 1.563, P< 0.001) and anxiety (OR = 1.452, P< 0.001). Compared to singles, married adults were less likely to report having depression (OR = 0.679, P = 0.007). While age was associated with increased risks for depression (OR = 1.186, P = 0.001) and anxiety (OR = 1.483, P ≤0.001), employment was shown to be protective against both disorders. The strongest link was found between depression and opium use (OR = 3.593, P< 0.001). BMI (OR = 1.392, P = 0.025), Alcohol consumption (OR = 1.969, P = 0.001), smoking (OR = 1.434, P = 0.006), and opioid use (OR = 2.556, P < 0.001) were all highly associated with risk of anxiety.

Both biological and social factors contribute to the increased incidence of anxiety and depression among women. For a variety of reasons, the psychological toll on women is often greater than that on men such as economic dependency, traditional gender roles, and social expectations. In addition, caregiving duties, gender-based violence, and hormonal changes may make women more susceptible to mental health issues.

Social isolation and financial instability put widows and the unemployeds in a precarious position. Living with the unfortunate circumstances of losing a spouse can lead to an array of mental issues such as depression and anxiety. With their condition exacerbated by the weak economy, the stress of not having a job can push these folks to the very edge. These results demonstrate the significance of financial stability in fostering mental health [5].

Given the concerning facts, targeted actions must be taken. Based on our findings, we recommend the following strategies:

1.Improved Screening and Diagnosis: Regular mental health screenings must to be a part of healthcare systems for adolescents and young adults. Early detection enables timely treatment and lowers the risk of long-term issues [6].

2.Comprehensive Mental Health Services: Therapies that treat substance misuse and mental health issues should be freely available, affordable, and culturally competent. For many populations, this ensures appropriate care [7].

3.Awareness Campaigns: Educational initiatives should highlight the risks associated with high-risk behaviors and their impact on mental health. Young people can make better life decisions when they are given information.

4.Support for Socioeconomic Stability: Programs that provide employment opportunities and provide financial assistance and Creating spaces for peer support and social interaction may reduce pressures that lead to mental health disorders [8][9].

The aforementioned tactics demonstrate how important it is to approach the problems brought up in this study from several perspectives. To create environments that prioritize mental health and resilience, community leaders, medical experts, and legislators must work together. They should ensure that mental health services are accessible for peoples who are at risk of mental issues, and organize themselves for early detection and intervention programs. Researchers should focus on longitudinal studies to track mental health trends and assess the effectiveness of interventions. Additionally, expanding cohort studies to different cultural and geographic contexts might help us better understand global mental health issues. Making mental health a public health priority requires constant commitment. Systemic changes that can be produced through collaborative efforts may lead to healthier prospects for younger generations.

### Ethical Statement

This study was approved by the Ethics Committee of Fasa University of Medical Sciences under the number IR.FUMS.REC.1402.070.

## Conflict of Interest

The authors declare that there is no conflict of interest associated with this research.
